# Lipoic Acid-Coated Silver Nanoparticles: Biosafety Potential on the Vascular Microenvironment and Antibacterial Properties

**DOI:** 10.3389/fphar.2021.733743

**Published:** 2022-01-28

**Authors:** Justyna Hajtuch, Maria Jose Santos-Martinez, Michal Wojcik, Ewelina Tomczyk, Maciej Jaskiewicz, Wojciech Kamysz, Magdalena Narajczyk, Iwona Inkielewicz-Stepniak

**Affiliations:** ^1^ Department of Pharmaceutical Pathophysiology, Medical University of Gdansk, Gdansk, Poland; ^2^ School of Pharmacy and Pharmaceutical Sciences and School of Medicine, Trinity Biomedical Sciences Institute, Trinity College Dublin, Dublin, Ireland; ^3^ Department of Organic Chemistry and Chemical Technology, Faculty of Chemistry, University of Warsaw, Warsaw, Poland; ^4^ Department of Inorganic Chemistry, Faculty of Pharmacy, Medical University of Gdansk, Gdansk, Poland; ^5^ Laboratory of Electron Microscopy, Faculty of Biology, University of Gdansk, Gdansk, Poland

**Keywords:** silver nanoparticles, biomaterial, lipoic acid coating, cytotoxicity, biocompatibility, endothelial cells, antimicrobial activity

## Abstract

**Purpose:** To study and compare the antibacterial properties and the potential cytotoxic effects of commercially available uncoated silver nanoparticles (AgNPs) with lipoic acid coated silver nanoparticles (AgNPsLA) developed by our group. The antibacterial, cytotoxic, and hemolytic properties of those NPs were assessed with the main objective of investigating if AgNPsLA could maintain their antibacterial properties while improving their biosafety profile over uncoated AgNPs within the blood vessel’s microenvironment.

**Methods:** Comercially available uncoated 2.6 nm AgNPs and 2.5 nm AgNPsLA synthesized and characterized as previously described by our group, were used in this study. Antimicrobial activity was assessed on a wide range of pathogens and expressed by minimal inhibitory concentrations (MIC). Assessment of cytotoxicity was carried out on human umbilical vein endothelial cells (HUVEC) using an MTT test. Detection of reactive oxygen species, cell apoptosis/necrosis in HUVEC, and measurement of mitochondrial destabilization in HUVEC and platelets were performed by flow cytometry. The potential harmful effect of nanoparticles on red blood cells (RBCs) was investigated measuring hemoglobin and LDH released after exposure to NPs. Transmission electron microscopy was also used to determine if AgNPs and AgNPsLA could induce any ultrastructural changes on HUVEC cells and *Staphylococcus aureus* bacteria*.*

**Results:** AgNPs and AgNPsLA had antimicrobial properties against pathogens associated with catheter-related bloodstream infections. AgNPs, in contrast to AgNPsLA, induced ROS production and apoptosis in HUVEC, ultrastructural changes in HUVEC and *S. aureus*, depolarization of mitochondrial membrane in HUVEC and platelets, and also hemolysis.

**Conclusion:** AgNPsLA synthesized by our group have antimicrobial activity and a better biosafety profile than uncoated AgNPs of similar size. Those observations are of critical importance for the future *in vivo* investigations and the potential application of AgNPsLA in medical devices for human use.

## Introduction

Nanomedicine is a field of science that applies the knowledge and tools of nanotechnology to the prevention and treatment of disease (Rani 2017). By the end of 2020 more than 200,000 scientific research articles were already published about nanomedicine (PubMed, 2020). Engineered nanoparticles (NPs) are endowed with exclusive characteristics, such as, among others, high surface area to volume ratio, high chemical reactivity, and even antimicrobial/fungicidal activity that makes them very attractive for their use in medicine (Medina et al., 2007). Modification of NPs surface may involve processes that can result in more desirable NPs properties, including reduction of cytotoxicity led by cellular events like oxidative stress and apoptosis (Abad et al., 2005). Turcu and co-workers (Turcu el al. 2017) developed LA-functionalized bioactive nanosystems using gold nanoparticles and demonstrated their biocompatibility at concentrations lower than 50 μg/ml using cell viability and cell cycle assays. Lipoic acid (LA) is a disulfide that it is reduced at intracellular levels into dihydrolipoic acid, a dithiol which has strong antioxidant properties, and it has been used for treating oxidative stress-related diseases like diabetic neuropathies (Salehi et al., 2019). Properties of LA including its metal chelating capacity, its ability to scavenge reactive oxygen species (ROS), its ability to regenerate endogenous antioxidants, and to repair oxidative damage have been demonstrated already. In fact, within the drug-related antioxidant pharmacology field, LA is a model compound that enhances understanding of the mode of action of antioxidants in drug therapy (Biewenga, Haenen, and Bast 1997).

It is estimated that up to 30% of all nosocomial bacteremias are associated with the use of intravascular devices such as central venous catheters with the involvement of strains such as *Enterococcus faecium, Staphylococcus aureus methicillin resistant, Klebsiella pneumoniae, Acinetobacter baumannii,* and *Pseudomonas aeruginosa* (Ruiz-Giardin et al., 2019). In addition, common complications associated with long-term use of central venous catheters are occlusions and catheter-related thrombosis. AgNPs are well known for having antimicrobial properties and unique characteristics that make them very attractive for biomedical applications (Hante et al., 2019). In one of our recent publications, we have demonstrated that AgNPsLA synthesized by our team are able to down-regulate P-selectin and GPIIb/IIIa receptor expression, inhibiting platelet aggregation under flow conditions. This effect is likely to be associated with thromboxane A2 formation and metalloproteinase release from platelets (Hajtuch et al., 2019). Based on these observations, it seems reasonable to think that, due to their anti-platelet and potential antibacterial properties, AgNPsLA could be useful to, for example, internally coat vascular catheters. However, it is necessary to accurately determine first if those NPs may also have some detrimental effects within the vascular microenvironment. The endothelium is particularly important for controlling macromolecules and fluid exchange between the blood and the interstitial space and it serves as a physiological physical barrier that controls the traffic of NPs from the vasculature into the surrounding tissue. In addition, the vascular endothelium is also involved in other physiological and pathological processes such as hemostasis and thrombosis, and inflammation and remodeling of the vascular wall (Engin et al., 2015). Changes in the mitochondrial membrane potential of platelets indicate mitochondria impairment. Mitochondria damage or dysfunction, as observed during several disease processes, results in attenuated platelet survival and increased risk for thrombovascular events (Melchinger et al., 2019). Red blood cells (RBCs) also play an important role within the circulating environment and the effect of NPs on them must be also investigated. In fact, although RBCs have been described as potential carriers of NPs for drug delivery, NPs may also have a deleterious effect inducing, for example, red cell lysis (Wadhwa et al., 2019).

In this work we aimed to investigate and compare the antibacterial properties and potential cytotoxic effects that commercially available bare silver nanoparticles (AgNPs) and lipoic acid coated AgNPsLA of similar size may exert within the blood vessel microenvironment. To this end, the effects of both types of nanoparticles on endothelial cells, platelets, and RBCs were examined. Moreover, their antimicrobial activity against a wide range of pathogens including those identified as etiological factors of potential catheter related infections was also investigated.

This study provides further support for the importance of the routine biosafety assessment of nanoparticles intended for future human applications and, in this particular case, for the potential use of those NPs as antiaggregating and antimicrobial agents for endoluminal catheter’s coatings.

## Materials and Methods

### Silver Nanoparticles

Uncoated silver nanoparticles (AgNPs) 2.6 nm size were purchased from United States Research Nanomaterials, Inc. (Houston, TX) and LA coated silver nanoparticles (AgNPsLA) 2.5 nm size synthesized by our group. Both types of NPs have been characterized and described in our previous research work (Zielinska et al., 2016; Zielinska et al., 2018; Hajtuch et al., 2019).

For measuring Ag release from the NPs, 1 ml sample of AgNPsLA and AgNPs (at concentrations of 10,000 and 2.000 μg/ml, respectively) were placed in a 3.5 kDa membrane and dialyzed in an external HUVEC culture medium (7 ml). During dialysis, 0.5 ml samples were taken at intervals of 1, 12, and 24 h from the media and subjected to ICP-MS analysis.

It is well known that NPs can interfere with *in vitro* methods that are commonly used for toxicological studies (Kroll et al., 2009; Liang et al., 2015). For this reason, NPs on their own and with the substrates used when relevant, where tested as an internal control in the assays involving the measurement of absorbance and during flow cytometry studies.

### Reference Strains of Microorganisms

Reference strains of bacteria that belong to ESKAPE pathogens (ESKAPE-bacterial pathogens commonly associated with antimicrobial resistance) were obtained from the American Type Culture Collection (ATCC); *Enterococcus faecium* (ATCC 700221), *Staphylococcus aureus methicillin resistant* (ATCC 33591), *Klebsiella pneumoniae* (ATCC 700603), *Acinetobacter baumannii* (ATCC 19606), *Pseudomonas aeruginosa* (ATCC 9027), and *Klebsiella aerogenes* (ATCC 13048) formerly *Enterobacter aerogenes* (ATCC 130480), and cultured following their recommendations. Briefly, all cultures were kept at −80°C using Roti^®^-Store Cryo-Vials (Carl Roth GmbH, Karlsruhe, Germany). Before the experiments, the cryo-protected bacteria were transferred into fresh Mueller-Hinton Broth media (Biocorp, Warsaw, Poland) and incubated for 24 h at 37°C on a rotating shaker (120 rpm). Cultures were then seeded on Mueller-Hinton Agarplates (Biocorp), incubated as mentioned above, and used for experiments. Cell densities for all assays were estimated and adjusted by optical density at 600 nm wavelength using a Multiskan™ 102 GO Microplate Spectrophotometer (Thermo Scientific).

### Determination of Antimicrobial Activity

Antimicrobial activity and minimal inhibitory concentrations (MICs) of the NPs tested were determined using the broth microdilution method according to Clinical and Laboratory Standards Institute (CLSI) guidelines. For this purpose, the initial inoculums of bacteria of 5 × 10^5^ CFU/ml in Mueller–Hinton Broth were exposed to AgNPs and AgNPsLA (0.5–256 μg/ml) and incubated for 18 h at 37°C. The experiments were conducted on 96-well polystyrene plates with a final volume of 100 μl. The MIC was taken as the lowest concentration at which a noticeable growth of microorganisms was inhibited. All experiments were conducted in triplicate.

### Cell Culture

Human umbilical vein endothelial cells (HUVEC) were obtained from Sigma Aldrich (cat number: 200P-05N) and maintained as a monolayer culture in T-75 cm^2^ tissue culture flasks. The tests were carried out in accordance with the manufacturer’s specifications, which recommends culture up to eight passages. HUVEC were cultured with endothelial cell growth medium (Sigma Aldrich 211–500) in the presence of antibiotics (6 μg/ml of penicillin-G, and 10 μg/ml streptomycin) at 37°C in a humidified atmosphere of 5% CO_2_. The medium was replaced every second day and when confluent, cells were detached with trypsin-EDTA and sub-cultured into new cell culture flasks.

### Cellular Experiments with Nanoparticles

For all experiments involving HUVEC, cells were co-incubated with AgNPs and AgNPsLA for 24 h. Concentrations used in those experiments varied and were determined from results obtained from preliminary studies. Just before being added to the cells, NPs were diluted in serum-free media and vortex for 1 min to ensure equal dispersion of NPs in the solution. Control cells were incubated with serum-free media in the absence of NPs. The medium was not changed during the 24 h of the incubation process.

### MTT Viability Assay

HUVEC were seeded in 96-well plates (15,000 cells per well) with complete media. After 24 h media was removed from the wells and cells were then co-incubated with NPs in a concentration range from 0.5 to 3.5 μg/ml for AgNPs or from 5 to 100 μg/ml for AgNPsLA. Following 24 h of incubation, the media was supplemented with water-soluble tetrazolium salt (at a final concentration of 0.5 μg/ml) and incubated for 2 h. Next, the media was removed, and the resultant crystals dissolved in DMSO. After 15 min, cell viability was assessed by measuring absorbance at 490 nm using a microplate reader (FLUOstar, OPTIMA). Viability was determined as a percentage of the control where the viability of the control cells was set as 100%. Absorbance values were corrected with blank NPs.

### Detection of Reactive Oxygen Species

HUVEC were seeded into 6-well plates; the next day, the medium was replaced and cells co-incubated with AgNPs at 0.5–3.5 μg/ml or with AgNPsLA at 25–100 μg/ml for 24 h. After the incubation time, media from each well was discarded and replaced with a new solution supplemented with 10 μM 2, 7-dichlorofluorescein diacetate (DCF-DA). After 30 min of incubation, the fluorescence of oxidized DCF was measured by flow cytometry (excitation wavelength: 480 nm; an emission wavelength: 525 nm) using a BD FACS Calibur. The data obtained for every sample was expressed as a percentage of the control (cells in the absence of NPs) and analyzed using the CellQuest software.

### Detection of Apoptosis/Necrosis

HUVEC were seeded into 6-well plates; the following day, the medium was replaced and cells were co-incubated with AgNPs at 1.5–3.5 μg/ml or with AgNPsLA at 25–100 μg/ml for 24 h. After the incubation time, cells were collected, washed twice with phosphate-buffered saline (PBS) (NaCl 0.138 M; KCl 0.0027 M; pH 7.4), and resuspended in binding buffer (50 mM HEPES: 4-(2-hydroxyethyl)-piperazineethanesulfonic acid, 700 mM NaCl, 12.5 mM CaCl_2_, pH 7.4). Afterward, 5 μl of Annexin V and 5 μl propidium iodine were added to the cells, gently resuspended once again, and incubated at room temperature in the dark for 15 min. Cells were then further diluted in binding buffer before flow cytometry analysis. Flow cytometry was carried out using a BD FACSArray (BD Biosciences, San Jose, CA) and 20,000 cell-specific events were analyzed for each experiment using the CellQuest software.

### Measurement of Mitochondrial Destabilization in Endothelial Cells and Platelets

HUVEC cells were seeded into 6-well plates; the next day, the medium was replaced, and cells treated with AgNPs at 1.5–3.5 μg/ml or with AgNPsLA at 25–100 μg/ml for 24 h. Afterward, the media for every well was discarded and replaced with a new solution supplemented with JC-1 (Cayman). Cells exposed to carbonyl cyanide 3-chlorophenylhydrazone (CCCP) were used as positive controls. In healthy cells with high mitochondrial membrane potential (ΔψM), JC-1 spontaneously forms complexes known as J-aggregates with intense red fluorescence. In case of apoptotic or unhealthy cells with low (ΔψM), JC-1 remains in the monomeric form, which shows only green fluorescence. The cells were detached, and 10,000 events counted by flow cytometry using a BD FACS Calibur. The fluorescence signals of JC-1 were detected in the fluorescence channels FL1. For microscope analysis, medium with JC-1 was replaced with fresh PBS and cells were observed under a fluorescence microscope (Olympus Life Science) and the data obtained analyzed using the CellQuest software.

To evaluate the potential mitochondrial destabilization in platelets, blood was withdrawn from healthy volunteers who had not been on any medication known to interfere with platelet function for at least 2 weeks prior to the study. The study was approved by the Bioethics Committee of the Medical University of Gdansk (NKBBN/552/2018-2019) and performed in accordance with the Code of Ethics of the World Medical Association, the ethical standards of the competent commission for Human Experiments (institutional and national) and the Helsinki Declaration of 1975 r., as amended in 2000. All volunteers gave informed consent before whole blood was collected and carefully mixed with 3.15% sodium citrate (9:1). Washed platelet (WP) suspensions were prepared from blood as described by Radomski and Moncada using Tyrode buffer at a final concentration of 250,000 platelets/μl (Radomski and Moncada 1983). Washed platelets were incubated with AgNPs or AgNPsLA for 1 h. After the incubation time, JC-1 was added, and 10,000 events measured by flow cytometry and the fluorescence signals of JC-1 were detected in the fluorescence channels FL1 (BD FACS Calibur) and data analyzed using the CellQuest software.

### Analysis of Hemolytic Properties of AgNPs and AgNPsLA

To assess the effect of NPs on RBCs, peripheral blood was collected into a syringe containing sodium citrate (0.35% final conc.) and 10 ml centrifuged at 300 xg for 10 min at room temperature. Platelet rich plasma and buffy coat were removed by aspiration and the red blood cells (RBCs) were diluted in PBS (1:100), plated in 96-wells and co-incubated with AgNPs or AgNPsLA for 12 h. Hemolysis was determined by measuring hemoglobin and LDH released (Promega Cytotox 96). Absorbance was recorded at 540 nm for hemoglobin and at 492 nm for LDH, using a microplate reader (FLUOstar, OPTIMA). Data were expressed as % of LDH release from RBCs according to the manufacturer’s instructions. For the data expressed as % of hemoglobin release, 0.1% Triton X -100 (Sigma-Aldrich, St. Louis, MO) was used as the positive control. One hundred percent lysis was corroborated by optical microscope.

### Analysis of Ultrastructural Changes by Transmission Electron Microscopy

Transmission electron microscopy (TEM) was used to investigate if AgNPs or AgNPsLA could induced ultrastructural changes in HUVEC and *S. aureus*. HUVEC were plated into 6-well plates. After 24 h cells were co-incubated with AgNPs (3.5 μg/ml) or AgNPsLA (50 μg/ml). *S aureus* (ATCC 33591) were grown overnight. Afterward, bacteria were centrifuged (1.000 xg, 7 min) and resuspended in fresh Mueller-Hinton medium to obtain a cell suspension of 5 × 10^5^ CFU/ml. Cells were then exposed to AgNPs or AgNPsLA at MIC concentrations and incubated for 18 h at 37°C. After treatment, bacteria were centrifuged (3.500 rpm, 10 min) and washed twice with PBS*.* HUVEC and *S. aureus* were fixed in 2.5% electron microscopy grade glutaraldehyde (Polysciences) in 0.1 M PBS (pH 7.4), post-fixed in 1% osmium tetroxide (Polysciences), dehydrated through a graded series of ethanol (30–100%), and embedded in Epon (Sigma). Ultrathin (65 nm) sections were cut using a Leica UC7 ultramicrotome, stained with Uranyless (Delta Microscopies) and Reynold’s lead citrate (Delta Microscopies), and examined on a Tecnai G2 Spirit BioTWIN TEM.

### Statistical Analysis

Data are expressed as mean ± SD of 3-4 independent experiments. The results are analyzed using one-way ANOVA and Tukey’s post hoc test, and a *p* value <0.05 is considered statistically significant. IC50 and logIC50 were also calculated using the GraphPad Prism 5 program V.5 (GraphPad Software Inc., San Diego, CA) by non-linear regression analysis.

## Results

### Silver Nanoparticles

AgNPs characterization data obtained previously confirmed stable and monodisperse negative charged NPs with sizes ranging from 1 to 5 nm with a mean diameter of 2.6 ± 0.8 nm by TEM EDS analysis in serum free media (Zielinska et al., 2016; Zielinska et al., 2018). In our previous study, we showed that our AgNPsLA are spherical with an average diameter of 2.5 ± 0.5 nm by TEM and SAXRD. The number of molecules attached to the NPs surfaces was determined by thermal gravimetric analysis (TGA) measurements. The % mass of organic was 41.31% and the number of ligands attached to the NPs was 176 (Hajtuch et al., 2019). ICP-MS analysis confirmed the stability of the AgNPsLA, as the silver content did not change significantly over time (0.015% after 24 h) when compared to the bare AgNPs that resulted in an increase in the content of silver ions over time in the HUVEC media (1.056% after 24 h) indicating a lower stability of these NPs under the conditions tested. Data are presented in Table 1.

**TABLE 1:** Characterization of AgNPs and AgNPsLA

Nanoparticles	Size/method	Number of ligands attached to the AgNPs/method	Silver content in medium (after 1 h) ICP-MS method	Silver content in medium (after 12 h) ICP-MS method	Silver content in medium (after 24 h) ICP-MS method
AgNPs	2.6 ± 0.8 nm	−	8.32 μg/ml	15.7 μg/ml	21.12 μg/ml
	TEM		0.416%	0.785%	1.056%
AgNPsLA	2.5 ± 0.5 nm	176	1 μg/ml	1.27 μg/ml	1.51 μg/ml
	TEM, SAXRD	TGA	0.01%	0.0127%	0.015%

a Based on results from our previous studies: Hajtuch et al., 2019; Zielinska et al., 2016; Zielinska et al., 2018).

### Antimicrobial Activity of AgNPs and AgNPsLA and Their Impact on Metabolic Activity in HUVEC

Table 2 shows the MIC of AgNPs and Ag NPsLA for *E. faecium, S. aureus, K. pneumonia, A. baumanii, P. aeuruginosa,* and *K. aerogenes*. Although AgNPs had a higher efficacy than AgNPsLA against most of the microorganisms, AgNPsLA were also active with the lowest minimal inhibitory concentrations (8 μg/ml) obtained against *A. baumani.*

**TABLE 2:** Minimal inhibitory concentrations of AgNPs and AgNPsLA against reference strains of microorganism

MIC (µg/ml)						
	*Enterococcus faecium*	*Staphylococcus aureus*	*Klebsiella pneumoniae*	*Acinetobacter baumanni*	*Pseudomonas aeruginosa*	*Klebsiella aerogenes*
AgNPs	4	16	8	8	4	4
AgNPsLA	32	32	32	8	32	32

**FIGURE 1:**
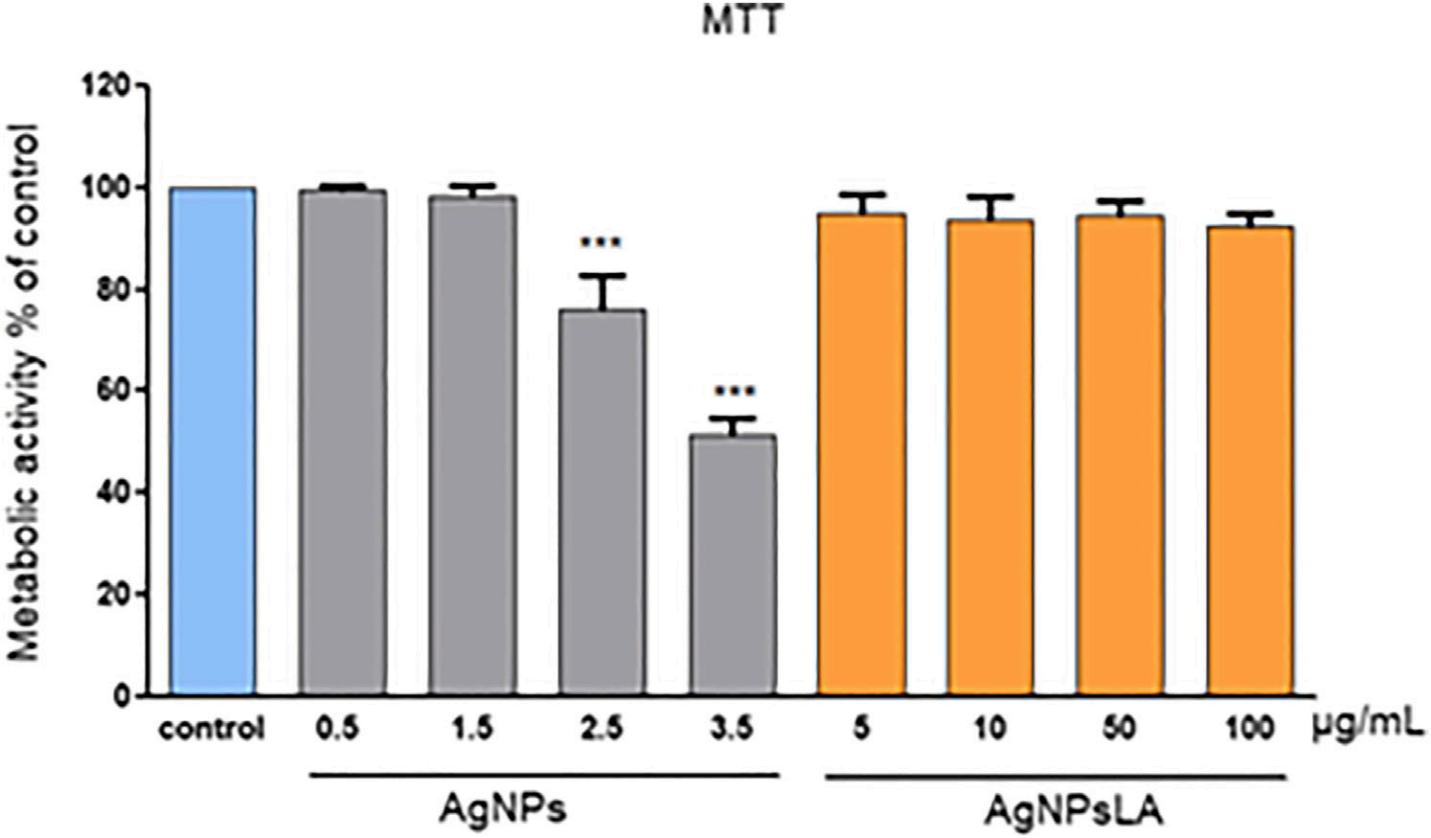
Metabolic activity of HUVEC cells as measured by MTT assay. Co-incubation of HUVEC cells for 24 h with AgNPsLA did not modify the metabolic activity of HUVEC cells. Exposure to bare AgNPs resulted in a significant decrease of their metabolic activity when compared to cells in the absence of NPs. Data are presented as mean ± SD. ***p < 0.001 vs. control.

Figure 1 illustrates the changes induced in the metabolic activity of HUVEC as measured by MTT assay after their exposure to AgNPs and AgNPsLA. AgNPs decreased the metabolic activity in the cells in a concentration equal or higher than 2.5 μg/ml. At the highest tested concentration (3.5 μg/ml), they decreased the metabolic activity of cells to around 50%. In contrast, AgNPsLA did not affect the metabolic activity of the cells at any of the concentrations tested.

The selectivity index (IC_50_/MIC) was also determined for AgNPs and AgNPsLA considering the average of MIC (X MIC) against *E. faecium, S. aureus, K. pneumonia, A. baumanii, P. aeuruginosa,* and *K. aerogenes,* and the IC_50_ on HUVEC (calculated based on the concentration of NPs that induced a 50% decrease of the metabolic activity in HUVEC as measured by the MTT assay). As shown in Table 3 the selectivity index (SI) of AgNPs and AgNPsLA values were 0.55 and 59.40, respectively.

**TABLE 3:** Selectivity index for AgNPs and AgNPsLA taking into account the average of MIC (X MIC) against *E. faecium, S.aureus, K. pneumonia, A. baumanii, P. aeuruginosa, K. aerogenes,* and the IC_50_ on HUVEC cells (SI = IC_50_/MIC) by the MTT assay

NPs	IC_50_ µg/ml	X MIC µg/ml	Selectivity index
AgNPs	3.5	6.35	0.55
AgNPsLA	1,509	25.40	59.40

### Impact of AgNPs and AgNPsLA on Reactive Oxygen Species Level

**FIGURE 2:**
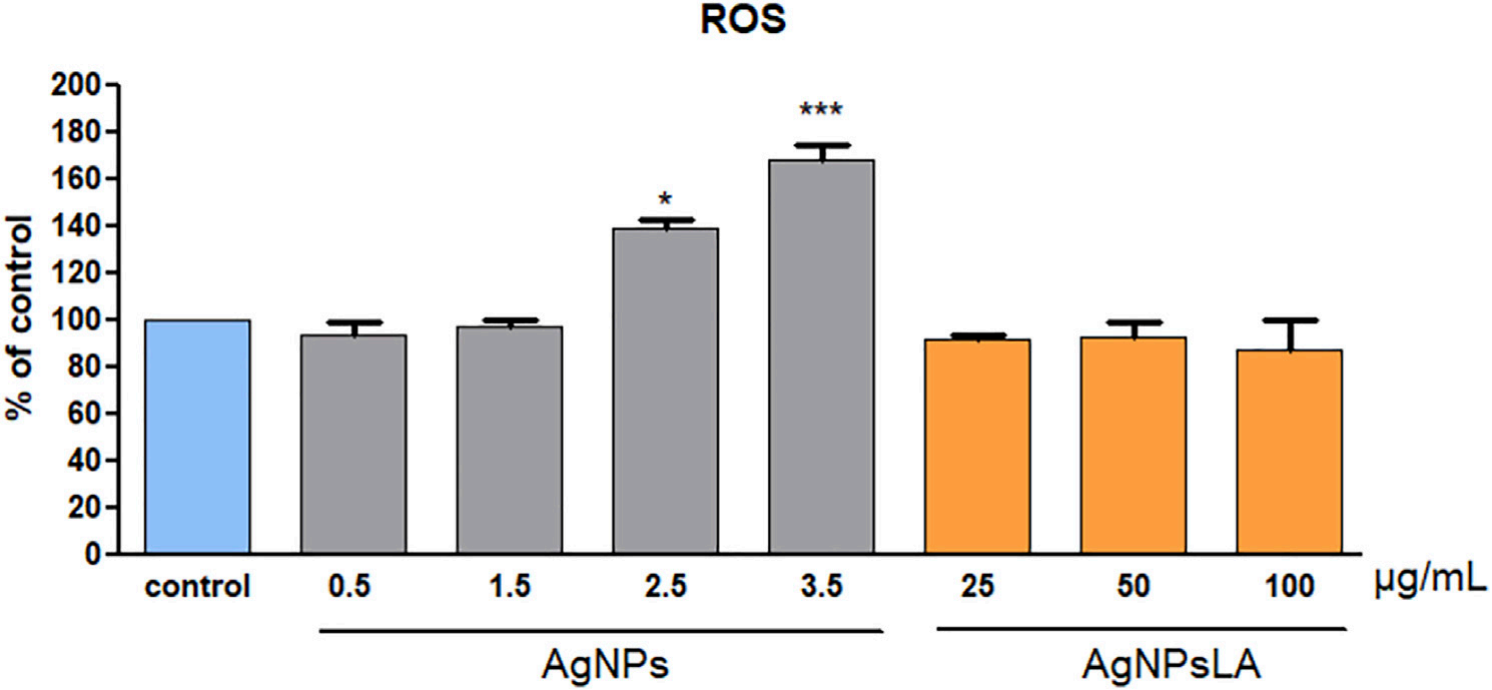
Reactive oxygen species (ROS) generation in HUVEC cells. Co-incubation of HUVEC cells for 24 h with AgNPsLA did not modify ROS generation in HUVEC cells. AgNPs induced a significant increase in ROS generation when compared to cells in the absence of NPs. Data are presented as mean ± SD. *p < 0.05, ***p < 0.001 vs. control.

As shown in Figure 2, AgNPs induced reactive oxygen species (ROS) generation in HUVEC in a concentration-dependent manner, namely 1.4 times (for 2.5 μg/ml) and 1.7 times (for 3.5 μg/ml) compared with non-treated cells. In contrast, AgNPsLA did not increase the ROS level in HUVEC compared with control cells after 24 h incubation.

### Impact of AgNPs and AgNPsLA on HUVEC Apoptosis

**FIGURE 3:**
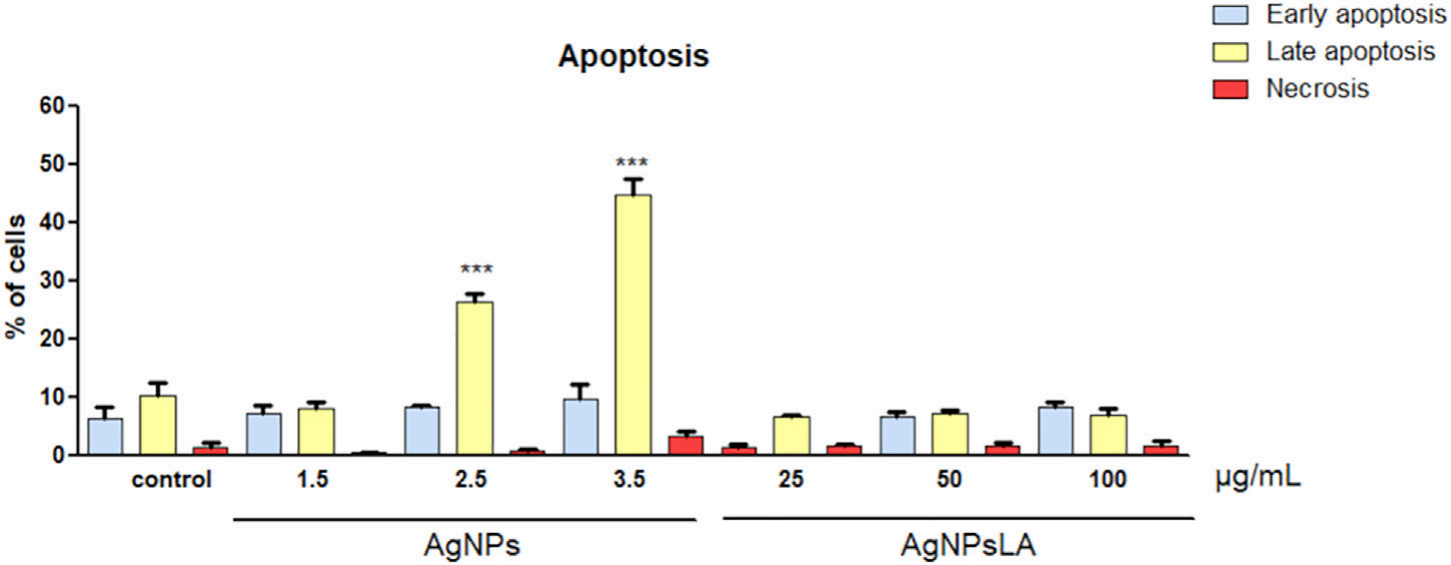
Effects on apoptosis in HUVEC cells. Apoptotic rates of HUVEC cells incubated with different concentrations of AgNPs and AgNPsLA for 24 h determined by flow cytometry. AgNPsLA had no effect, AgNPs induced late apoptosis at the highest concentrations tested. Data are presented as mean ± SD of three independent experiments. ***p < 0.001 vs. untreated cells.

Next, we determined the type of cell death by dual staining of HUVEC with propidium iodide (PI) and Annexin V, which allows us to distinguish between the amount of early apoptotic (Annexin V^+^ PI^−^), late apoptotic/necroptotic (Annexin V^+^ PI^+^), and necrotic (Annexin V^−^ PI^+^) cell populations (Lakshmanan 2016). After 24 h of incubation with AgNPs, a significant increase rate of late apoptotic population of HUVEC cells up to 28 and 45% was detected for 2.5 μg/ml and 3.5 μg/ml, respectively. In contrast, AgNPsLA caused neither apoptosis nor necrosis in HUVEC within the range of the investigated concentrations (Figure 3).

### Impact of AgNPs and AgNPsLA on Mitochondrial Depolarization

**FIGURE 4:**
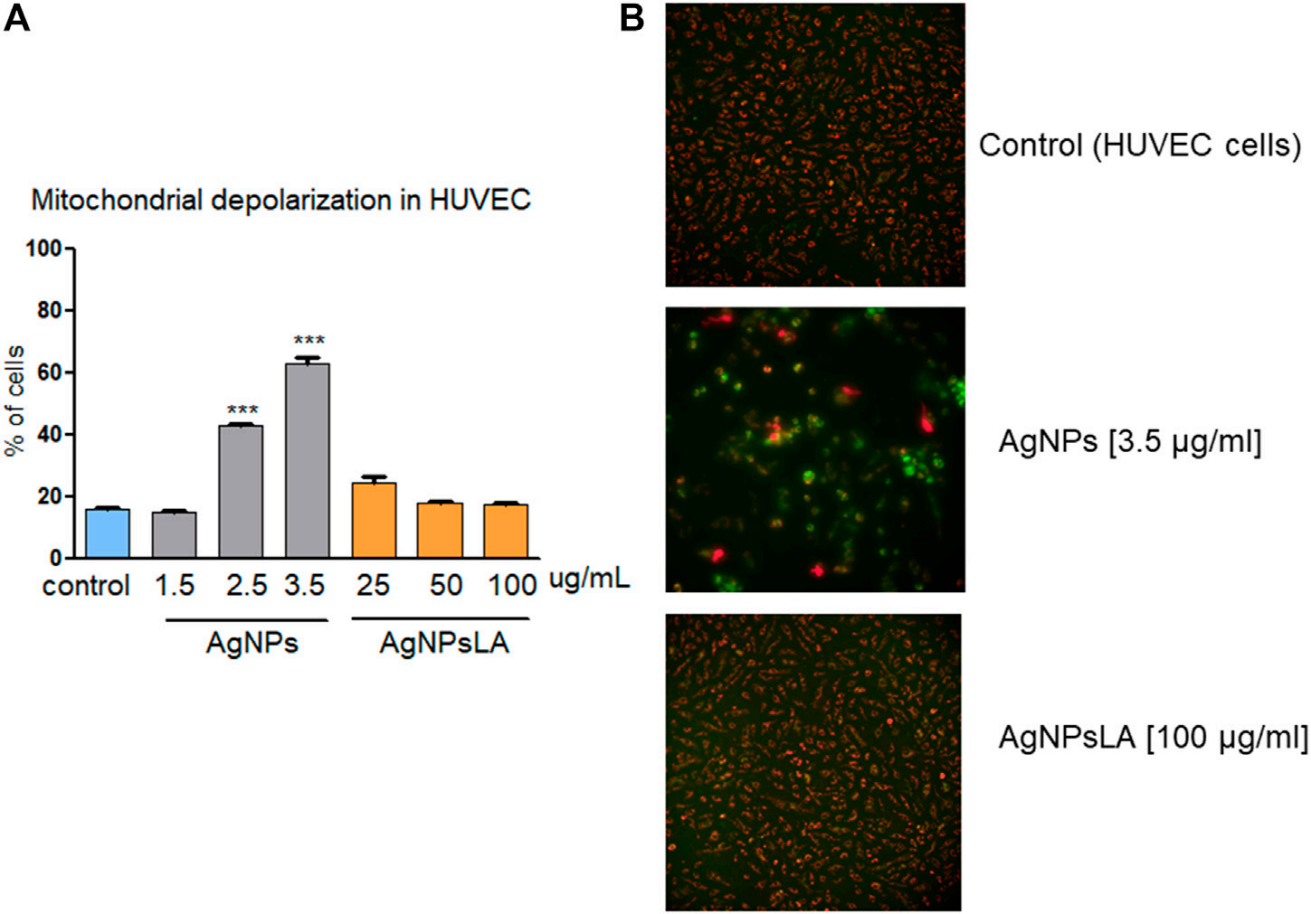
Mitochondria depolarization in HUVEC. AgNPs induced mitochondria damage in HUVEC, no changes in membrane potential were detected in cells treated with AgNPsLA. **(A)** Percentage of cells associated to decreased mitochondrial membrane depolarization. Data are presented as mean ± SD of three independent experiments. ***p < 0.001 vs. control. **(B)** Representative images of JC-1 staining in HUVEC in the presence and absence of AgNPs and AgNPsLA. Red fluorescence is formed by JC-1 complexes in undamaged mitochondria, whereas green fluorescence is formed by JC-1 monomers in mitochondria with low membrane potential.

**FIGURE 5:**
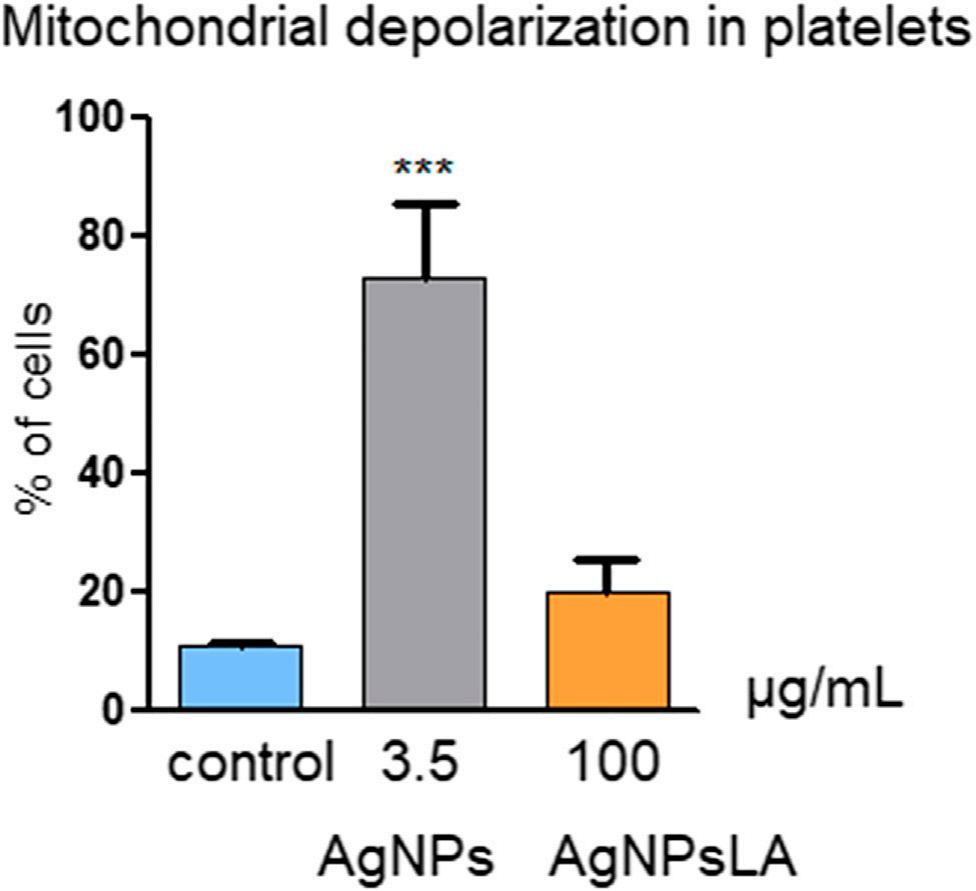
Mitochondria depolarization in platelets. AgNPs caused significant changes in membrane potential of human platelets, in contrast to AgNPsLA. Percentage of cells with decreased mitochondrial membrane depolarization. Data are presented as mean ± SD of three independent experiments. ***p < 0.001 vs. control.

Mitochondria play an essential role in activating caspase proteases through a pathway termed the mitochondrial or intrinsic pathway of apoptosis (Tait and Green 2013). To confirm further that AgNPs induce apoptosis, mitochondrial membrane potential was measured in HUVEC following incubation with AgNPs and AgNPsLA. JC-1 staining was used to determine NPs impact on mitochondria. Figure 4 shows that AgNPs induced a significant damage to mitochondria after 24 h incubation, but no changes in membrane potential were detected in cells treated with AgNPsLA. The highest concentrations of AgNPs tested (2.5 and 3.5 μg/ml) significantly increased the polarization of mitochondrial membrane in HUVEC. Similarly, AgNPs also induced changes in membrane potential of human platelets, in contrast to AgNPsLA (Figure 5).

### Evaluation of Hemolytic Activity of AgNPs and AgNPsLA

**FIGURE 6:**
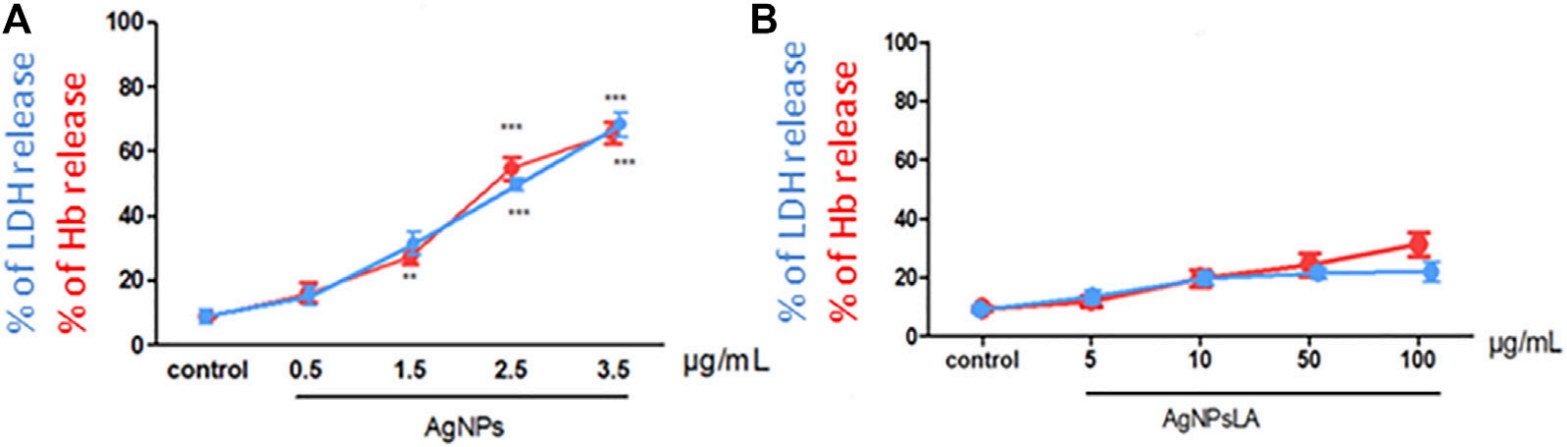
Effect of AgNPs and AgNPsLA on red blood cells (RBCs). **(A)** % of LDH release (blue line) and % of hemoglobin (Hb) release (red line) from RBCs after incubation with AgNPs. **(B)** % of LDH release (blue line) and % Hb release (red line) from RBCs AgNPsLA treated. Data are presented as mean ± SD. **p < 0.01 ***p < 0.001 vs. control.

To evaluate the potential deleterious effect of AgNPs and AgNPsLA on RBCs, hemoglobin (Hb) and LDH release from RBCs in the presence of both NPs were measured. AgNPs led to concentration dependent hemolysis reflected by ∼65% of Hb and ∼80% of LDH release at the concentration of 3.5 μg/ml AgNPs (Figure 6A). In contrast, AgNPsLA did not induce significant hemolysis leading to ∼30% of Hb and ∼20% of LDH release at the highest concentration tested (100 μg/ml) (Figure 6B).

### Impact of AgNPs and AgNPsLA on Ultrastructural Changes

**FIGURE 7:**
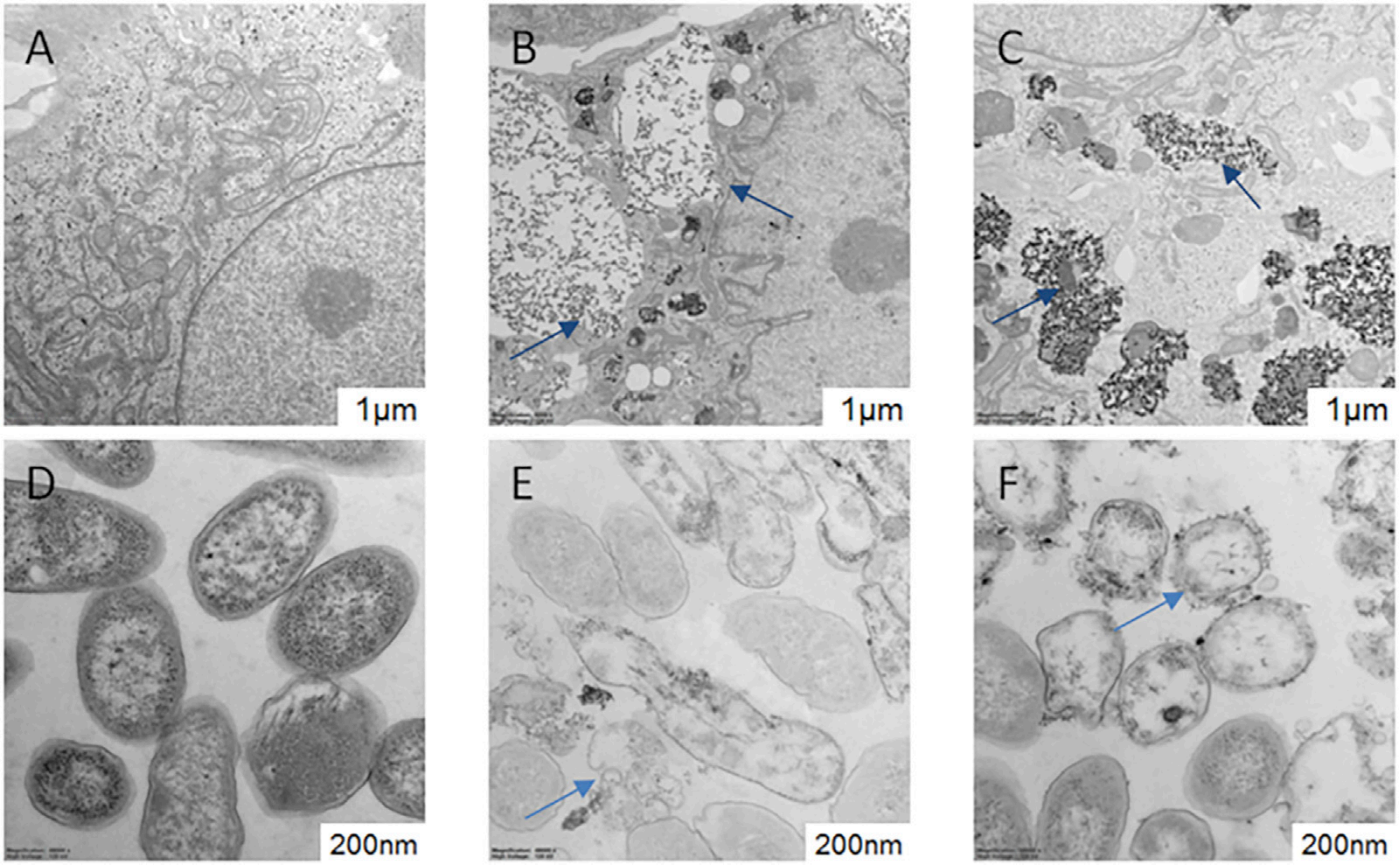
TEM of HUVEC and *Staphylococcus aureus* morphology. **(A)** HUVEC control cells; **(B)** HUVEC treated with AgNPs (3.5 μg/ml). Blue arrows indicate AgNPs in autolysosomes and vesicles. **(C)** HUVEC treated with AgNPsLA (50 μg/ml). Blue arrows indicate AgNPsLA. **(D)** *S. aureus*. Control. **(E)** *S. aureus* treated with AgNPs (16 μg/ml). **(F)** *S. aureus* treated with AgNPsLA (32 μg/ml). The blue arrows point the nanoparticles.

TEM analysis of HUVEC in the absence and presence of AgNPs and AgNPs is shown in Figures 7A–C. TEM images show the presence of both tested NPs located mainly in autolysosomes and vesicles. The morphology and structure of *S. aureus* were also examined by TEM after their exposure to AgNPs and AgNPsLA (Figures 7D–F). In contrast to unexposed bacteria, those incubated with both types of nanoparticles (AgNPs and AgNPsLA) showed structural changes mainly in the cell wall, which became thin, wrinkled, and sometimes broken. In addition, some bacteria showed signs of swelling or atrophy combined with deformation or rupture of the cell membrane and release of the cell contents.

## Discussion

Nowadays, development of novel, efficient nanotechnological-based bactericidal agents against multidrug-resistant bacteria is among one of the priority areas in biomedical research (Dakal et al., 2016). *In vitro* studies available in the literature testing the effect of AgNPs have reported that they can be toxic to several human cell lines. Furthermore, and based on already published *in vivo* animal models, it is known that AgNPs tend to accumulate in the liver, spleen, and kidney and that they are able to cross the blood-brain barrier following intravenous, intraperitoneal, and intratracheal routes of administration (Liao et al., 2019). In this study, we assessed the biosafety of bare AgNPs and AgNPs coated with LA on the vascular microenvironment as they could be potentially used to coat medical devices such as vascular catheters.

The AgNPsLA synthesized by our team (2.5 nm size) demonstrated a fairly constant silver content over time in the media, while the commercially available AgNPs (2.6 nm size) that we used for this study showed an increase in the silver ion content over time, indicating a lower stability of these NPs. However, in our previous research, the soluble Ag present in the serum free medium after 24 and 48 h of exposure to 15 nm AgNPs was found to correspond to a release of less than 0.5% (Zielinska et al., 2016). Research data available in the literature indicate a correlation between the physicochemical behavior of AgNPs in cell culture medium or in a simulated biological medium with their toxicity toward cultured cells. In fact, the higher the *in vitro* silver release, the higher the toxicity of the AgNPs. In addition, coating of AgNPs can reduce the dissolution of silver particles in water for several days mitigating as well silver release (Veronesi et al., 2016; Cunningham et al., 2021; Zhang et al., 2021). We demonstrate that although the bare AgNPs used in this study induce cytotoxicity and hemolysis in a concentration-dependent manner, co-incubation with AgNPs coated with LA do not exert harmful effects on HUVEC, platelets, and RBCs. Moreover, those AgNPsLA have antimicrobial properties at concentrations that are safe for them.

### AgNPs and AgNPsLA: Antimicrobial Properties and Metabolic Activity in HUVEC

With the emergence of pathogenic bacterial strains resistant to one or several antibiotics, there is an urgent need for new antibacterial agents. It has been demonstrated that AgNPs action is highly dependent on their surface reactivity (Le Ouay and Stellacci 2015). In our hands, bare AgNPs exhibited higher antimicrobial activity than AgNPsLA. We focused on six pathogens: *E. faecium, S. aureus, K. pneumonia, A. baumanii, P. aeuruginosa, and K. aerogenes*. Selected pathogens are well known as etiological factors of catheter related infections (Bouza et al., 2002; Shah et al., 2013). Many research studies have previously shown the antimicrobial activity of uncoated AgNPs (Kim et al., 2007; Qing et al., 2018; Prakash et al., 2019). However, when it comes to LA coated NPs, only a few studies have examined their antibacterial properties (Cotton et al., 2019). Our team, has previously shown that AgNPsLA at concentrations ≤ 5–40 μg/ml inhibited growth of 70% of the tested bacteria (Niska et al., 2016). Our LA coated NPs exhibited a MIC range of 8–32 μg/ml for the reference strains.

Isolation of human umbilical vein endothelial cells (HUVEC) from umbilical cord was first described in 1973. To date, this model is still widely used due to the high success rate of HUVEC isolation and because this is an excellent model for studying a wide range of diseases, including cardiovascular and metabolic diseases. The vascular endothelium plays a crucial role in the regulation of blood pressure, blood flow, and coagulation (Leyte et al., 2020). The HUVEC model is in fact highly representative of the human vascular endothelium, allowing the study of the physiological and pathological effects of different factors (Maciag et al., 1981). We have previously demonstrated that AgNPsLA inhibit platelet aggregation (Hajtuch et al., 2019). So, based on our previous findings we hypothesized that those NPs could be used to internally coat an intravenous catheter for preventing thrombosis but to also fight against associated microbial infections. For this reason, we have focused on assessing further the potential effect that those NPs could exert not only on platelets but also on endothelial cells and red blood cells.

The MTT assay estimates cell viability by measuring mitochondrial metabolism. The enzymatic reduction of 3-[4,5-dimethylthiazole-2-yl]-2,5-diphenyltetrazolium bromide (MTT) to MTT-formazan is catalyzed by mitochondrial succinate dehydrogenase. Hence, the MTT assay is dependent on mitochondrial respiration and indirectly serves to assess the cellular energy capacity of a cell (Chacon et al., 1997). Our results showed that although bare AgNPs exerted cytotoxic effects on HUVEC, AgNPsLA did not lead to toxicity even at the highest concentration tested (100 μg/ml). Those results are in agreement with our previous work where we demonstrated a lower cytotoxicity of LA coated AgNPs when compared to bare AgNPs using a gingival fibroblast model where AgNPsLA did not induce cell toxicity at concentrations up to 40 μg/ml (Niska et al., 2016). Selectivity index is the ratio that measures the window between cytotoxicity and antibacterial activity. The higher the SI index, the more effective and safer the material would be during *in vivo* treatment. Therefore, an ideal material should be cytotoxic only at very high concentrations but should show antibacterial activity at very low concentrations, which would result in a high SI value (Pritchett et al., 2014). In our research, SI of AgNPs and AgNPsLA values were 0.55 and 59.40, respectively, indicating the effectiveness and safety of AgNPsLA as a potential biomaterial.

### Functionalization (Coating) With Lipoic Acid Prevents AgNPs-Induced ROS Production, Apoptosis and Mitochondrial Depolarization

ROS play a central role in cell signaling as well as in regulation of the main pathways of apoptosis mediated by mitochondria. Apoptosis is a tightly regulated and highly conserved process of cell death during which a cell undergoes self-destruction (Redza-Dutordoir and Averill-Bates 2016). Mitochondrial dysfunction has been shown to participate in the induction of apoptosis and has even been suggested to be central to the apoptotic pathway. Indeed, opening of the mitochondrial permeability transition pore has been demonstrated to induce depolarization of the transmembrane potential, release of apoptogenic factors, and loss of oxidative phosphorylation (Gottlieb et al., 2003). Hence, we aimed to examine the impact of bare AgNPs and AgNPsLA on ROS, apoptosis, and mitochondrial membrane potential. BareAgNPs significantly increased ROS production, apoptosis, and mitochondrial depolarization, when compared to control and to AgNPsLA incubated cells. In this study, we have found that AgNPs induced mitochondrial membrane depolarization suggesting the formation of the mitochondrial permeability transition pore transmembrane protein in AgNPs incubated cells, which is consistent with ROS role in triggering cell damage processes including the formation of mitochondrial permeability transition pore (AshaRani et al., 2009). Many other studies have also reported the generation of ROS in different cell lines in the presence of AgNPs, which are in agreement with our results (Barcińska et al., 2018; El-Hussein and Hamblin 2018; Kang et al., 2012). However, we have reported for the first time that LA coating prevents the increase in ROS induced by bare AgNPs in HUVEC and that AgNPsLA do not affect the mitochondrial membrane potential of platelets, something that is of crucial importance. In fact, platelets do not have nuclei and therefore their function and span life largely depend on the functioning of their mitochondria (Melchinger et al., 2019) and the mitochondrial membrane potential of human platelets is considered a sensitive parameter of platelet quality (Verhoeven et al., 2005).

### Impact of AgNPs and AgNPsLA on RBCs

Hemolysis refers to the breakdown of erythrocytes with subsequent release of their intracellular content. This can result in a dangerous situation that can ultimately lead to jaundice and severe anemia. Many natural and synthetic nanoparticles have elicited hemolytic action and hence, preclinical investigation of hemolytic activity is of massive importance for newly developed nanomedicines for human use that may come in contact with blood (Raval et al., 2018). Lactate dehydrogenase (LDH) can be physiologically present in serum due to cellular turnover but during hemolytic conditions LDH is often increased (Barcellini and Fattizzo 2015). Our results clearly show that bare AgNPs can induce hemolysis as demonstrated by an increase of hemoglobin and LDH release from erythrocytes co-incubated with those NPs. In fact, it has been previously found that AgNPs have hemolytic effects by changing membrane integrity and surface characteristics and that they might cause pore formation on the membrane of RBCs that ultimately results in osmotic lysis (Kwon et al., 2012; Krajewski et al., 2013). It has been described (Uchendu et al. (2014) that pretreatment with LA decreases erythrocyte fragility due to lipid peroxidation. Interestingly, the AgNPsLA tested in our work did not induce significant hemolysis when incubated with RBCs.

### Impact of AgNPs and AgNPsLA on Ultrastructure of HUVEC and *S. aureus*

TEM analysis demonstrated that the exposure of HUVEC cells to AgNPs and AgNPsLA led to NPs accumulation in autolysosomes and vesicles, which is consistent with the earlier study published by Guo et al. (2016) that demonstrated AgNPs uptake by HUVEC cells. Moreover, both AgNPs and AgNPsLA significantly affect the cell membrane integrity when co-incubated with *S. aureus*. Those experiments confirmed the presence of cell wall damage caused by those NPs, which is the basic mechanism of antibacterial action, as well as the accumulation of NPs in the bacterial membrane (Mirzajani et al., 2011; Bondarenko et al., 2018).

### Research Limitations

Most experiments for characterizing the biological effects of nanomaterials are performed *in vitro* using cell lines. In fact, their potential toxicological effect, uptake mechanisms as well as other specific cellular responses, such as the measurements of ROS, can be carried out in different cell lines. However, one limitation of this approach is that the absorption and viability results recorded *in vitro* may differ from those observed in more organized systems, such as tissues or organs, or *in vivo* (Bakand and Hayes 2016). Under physiological conditions, endothelial cells are constantly exposed to shear stresses induced by blood flow and its viscosity, and the responses of these cells to stimuli may be determined by shear stress patterns. Indeed, endothelial cells are protected during laminar flow against harmful stimuli, while uneven shear stress can activate the endothelium (Matuszak et al., 2016). Recent studies indicate that endothelial uptake by non-targeted NPs depends on the presence and magnitude of shear stress (Fede et al., 2015; Klingberg et al., 2015). Therefore, and compared to traditional *in vitro* cytotoxicity assays performed under static conditions, NPs may exhibit higher and stress-dependent toxicity to endothelial cells under flow conditions (Shurbaji et al., 2020). It is worth to mention that advanced culture systems are expensive, technically demanding, and more difficult to standardize and that it is currently unclear to what extent advanced culture systems provide more predictive data on human toxicity than conventional systems. However, databases based on results obtained from conventional cell cultures, advanced models and animal experiments are crucial for defining the role of advanced culture systems in the toxicological assessment of NPs (Fröhlich 2018). There is no doubt that future research using models that mimic more closely physiological conditions, like in this particular case flow-through experiments, would provide a better inside on the effect of AgNPsLA on HUVEC cells under near-physiological conditions.

## Conclusion

We compared the antimicrobial effect and potential deleterious effect that bare AgNPs and LA-coated AgNPs may had on endothelial cells, platelets, and RBCs. Co-incubation of RBC led to hemolysis with AgNPs but not with AgNPsLA. While considerable cytotoxicity was demonstrated in HUVEC cells for bare AgNPs at low concentrations (2.5 μg/ml), AgNPsLA did not affect cell viability of HUVEC when they were co-incubated up to 100 μg/ml for 24 h. Cell death associated to AgNPs incubations was found to be a ROS-dependent apoptosis mechanism accompanied by a depolarization of the mitochondrial membrane potential. In contrast, we showed, for the first time, that functionalization of AgNPs with LA maintains the antimicrobial activity associated and widely demonstrated for AgNPs but significantly reduces NPs cytotoxicity on endothelial, platelets, and red blood cells. Our work supports once again the importance for carrying out biocompatibility studies when investigating the potential application of nanotechnology in medicine.

**FIGURE 8:**
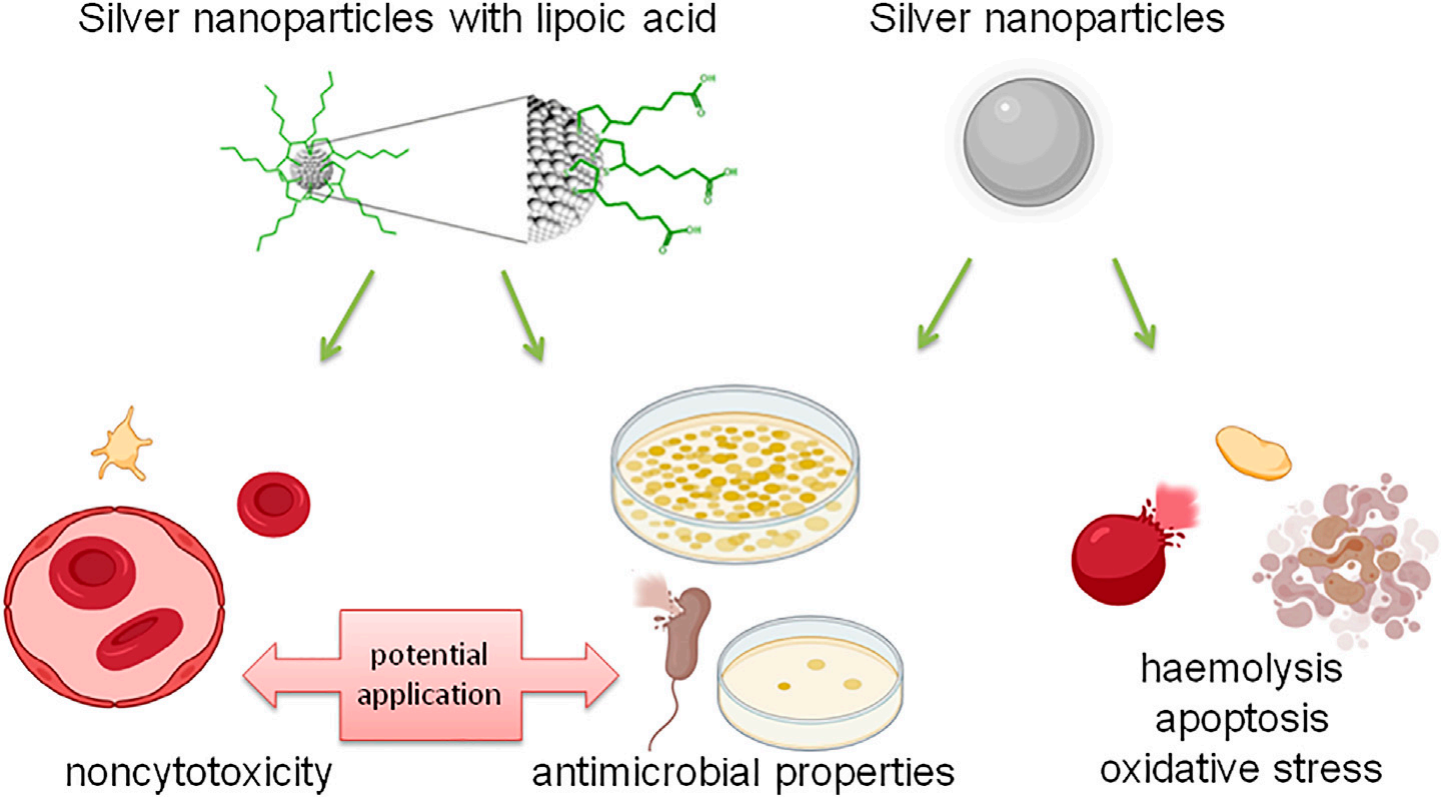
Summary of the assessment of antibacterial properties and the potential of biosafety in the vascular microenvironment of new lipoic acid coated AgNPs and uncoated AgNPs.

Figure 8 summarizes assessment of the antibacterial properties and biosafety potential on the vascular microenvironment of novel lipoic acid-coated AgNPs and uncoated AgNPs.

## Data Availability

The original contributions presented in the study are included in the article/Supplementary Material. Further inquiries can be directed to the corresponding author.

## References

[B1] Abad J. M. Mertens S. F. Pita M. Fernández V. M. Schiffrin D. J. (2005). Functionalization of Thioctic Acid-Capped Gold Nanoparticles for Specific Immobilization of Histidine-Tagged Proteins. J. Am. Chem. Soc. 127 (15), 5689–5694. 10.1021/ja042717i 15826209

[B2] Ahmed El H. Hamlin M R. (2018). ROS Generation and DNA Damage with Photo-Inactivation Mediated by Silver Nanoparticles in Lung Cancer Cell Line. IET Nanobiotechnol 176 (1), 139–148. 10.1016/j.physbeh.2017.03.040 PMC5505734 28477000

[B3] AshaRani P. V. Hande M. P. Valiyaveettil S. (2009). Anti-Proliferative Activity of Silver Nanoparticles. BMC Cel Biol 10, 65. 10.1186/1471-2121-10-65 PMC2759918 19761582

[B4] Bakand S. Hayes A. (2016). Toxicological Considerations, Toxicity Assessment, and Risk Management of Inhaled Nanoparticles. Int. J. Mol. Sci. 17 (6), 1–17. 10.3390/ijms17060929 PMC4926462 27314324

[B5] Barcellini W. Fattizzo B. (2015). Clinical Applications of Hemolytic Markers in the Differential Diagnosis and Management of Hemolytic Anemia. Dis. Markers 2015, 1–7. 10.1155/2015/635670 PMC4706896 26819490

[B6] Barcińska E. Wierzbicka J. Zauszkiewicz-Pawlak A. Jacewicz D. Dabrowska A. Inkielewicz-Stepniak I. (2018). Role of Oxidative and Nitro-Oxidative Damage in Silver Nanoparticles Cytotoxic Effect against Human Pancreatic Ductal Adenocarcinoma Cells. Oxidative Med. Cell Longevity 2018, 1–15. 10.1155/2018/8251961 PMC6116403 30186549

[B7] Biewenga G. P. Haenen G. R. Bast A. Bast Aalt. (1997). The Pharmacology of the Antioxidant Lipoic Acid. Gen. Pharmacol. 29 (3), 315–331. 10.1016/S0306-3623(96)00474-0 9378235

[B8] Bondarenko O. M. Sihtmäe M. Kuzmičiova J. Ragelienė L. Kahru A. Daugelavičius R. (2018). Plasma Membrane Is the Target of Rapid Antibacterial Action of Silver Nanoparticles in Escherichia Coli and Pseudomonas Aeruginosa. Int. J. Nanomedicine 13, 6779–6790. 10.2147/IJN.S177163 30498344 PMC6207270

[B9] Bouza E. Burillo A. Muñoz P. (2002). Catheter-Related Infections: Diagnosis and Intravascular Treatment. Clin. Microbiol. Infect. 8 (5), 265–274. 10.1046/j.1469-0691.2002.00385.x 12047403

[B10] Chacon Enrique. Acosta Daniel. Lemasters John. J. (1997). “Primary Cultures of Cardiac Myocytes as *In Vitro* Models for Pharmacological and Toxicological Assessments,” *in Vitro* Methods in Pharmaceutical Research: Pharmacotoxicological Effects of Drugs (Cambridge, Massachusetts: Academic Press). 10.1016/b978-012163390-5.50010-7

[B11] Cotton G. C. Gee C. Jude A. Duncan W. J. Abdelmoneim D. Coates D. E. (2019). Efficacy and Safety of Alpha Lipoic Acid-Capped Silver Nanoparticles for Oral Applications. RSC Adv. 9 (12), 6973–6985. 10.1039/c9ra00613c PMC9061105 35518463

[B12] Cunningham B. Engstrom A. M. Harper B. J. Harper S. L. Mackiewicz M. R. (2021). Silver Nanoparticles Stable to Oxidation and Silver Ion Release Show Size-dependent Toxicity *In Vivo* . Nanomaterials (Basel) 11 (6), 1516. 10.3390/nano11061516 34201075 PMC8230025

[B13] Dakal T. C. Kumar A. Majumdar R. S. Yadav V. (2016). Mechanistic Basis of Antimicrobial Actions of Silver Nanoparticles. Front. Microbiol. 7 (NOV), 1–17. 10.3389/fmicb.2016.01831 27899918 PMC5110546

[B14] Engin E. A. Neagu M. Golokhvast K. Tsatsakis A. (2015). Nanoparticles and Endothelium: An Update on the Toxicological Interactions. Farmacia 63 (6), 792–804.

[B15] Fede C. Fortunati I. Weber V. Rossetto N. Bertasi F. Petrelli L. (2015). Evaluation of Gold Nanoparticles Toxicity towards Human Endothelial Cells under Static and Flow Conditions. Microvasc. Res. 97, 147–155. 10.1016/j.mvr.2014.10.010 25446009

[B16] Fröhlich E. (2018). Comparison of Conventional and Advanced *In Vitro* Models in the Toxicity Testing of Nanoparticles. Artif. Cell Nanomedicine, Biotechnol. 46 (Suppl. 2), 1091–1107. 10.1080/21691401.2018.1479709 PMC6214528 29956556

[B17] Gottlieb E. Armour S. M. Harris M. H. Thompson C. B. (2003). Mitochondrial Membrane Potential Regulates Matrix Configuration and Cytochrome C Release during Apoptosis. Cell Death Differ 10 (6), 709–717. 10.1038/sj.cdd.4401231 12761579

[B18] Guo H. Zhang J. Boudreau M. Meng J. Yin J. J. Liu J. (2016). Intravenous Administration of Silver Nanoparticles Causes Organ Toxicity through Intracellular ROS-Related Loss of Inter-endothelial junction. Part. Fibre Toxicol. 13 (1), 21–13. 10.1186/s12989-016-0133-9 27129495 PMC4850669

[B19] Hajtuch J. Hante N. Tomczyk E. Wojcik M. Radomski M. W. Santos-Martinez M. J. (2019). Effects of Functionalized Silver Nanoparticles on Aggregation of Human Blood Platelets. Int. J. Nanomedicine 14, 7399–7417. 10.2147/IJN.S213499 31571858 PMC6750026

[B20] Hante N. K. Medina C. Santos-Martinez M. J. Jose Santos-Martinez Maria. (2019). Effect on Platelet Function of Metal-Based Nanoparticles Developed for Medical Applications. Front. Cardiovasc. Med. 6 (September), 139. 10.3389/fcvm.2019.00139 31620449 PMC6759469

[B21] Kang K. Jung H. Lim J. S. (2012). Cell Death by Polyvinylpyrrolidine-Coated Silver Nanoparticles Is Mediated by ROS-dependent Signaling. Biomol. Ther. (Seoul) 20 (4), 399–405. 10.4062/biomolther.2012.20.4.399 24009827 PMC3762268

[B22] Kim J. S. Kuk E. Yu K. N. Yu. Kim J.-H. ParkLee S. J. Lee H. J. (2007). Antimicrobial Effects of Silver Nanoparticles. Nanomedicine: Nanotechnology, Biol. Med. 3 (1), 95–101. 10.1016/j.nano.2006.12.001 17379174

[B23] Klingberg H. Loft S. Oddershede L. B. Møller P. (2015). The Influence of Flow, Shear Stress and Adhesion Molecule Targeting on Gold Nanoparticle Uptake in Human Endothelial Cells. Nanoscale 7 (26), 11409–11419. 10.1039/c5nr01467k 26077188

[B24] Krajewski S. Prucek R. Panacek A. Avci-Adali M. Nolte A. Straub A. (2013). Hemocompatibility Evaluation of Different Silver Nanoparticle Concentrations Employing a Modified Chandler-Loop *In Vitro* Assay on Human Blood. Acta Biomater. 9 (7), 7460–7468. 10.1016/j.actbio.2013.03.016 23523936

[B25] Kroll A. Pillukat M. H. Hahn D. Schnekenburger J. (2009). Current *In Vitro* Methods in Nanoparticle Risk Assessment: Limitations and Challenges. Eur. J. Pharm. Biopharm. 72 (2), 370–377. 10.1016/j.ejpb.2008.08.009 18775492

[B26] Kwon T. WooPark H. J. KimPark Y. H. Lee H. J. Park K. H. Park S. (2012). Optimizing Hemocompatibility of Surfactant-Coated Silver Nanoparticles in Human Erythrocytes. J. Nanosci Nanotechnol 12 (8), 6168–6175. 10.1166/jnn.2012.6433 22962723

[B27] Lakshmanan I. BatraBatra S. K. (2016). Protocol for Apoptosis Assay by Flow Cytometry Using Annexin V Staining Method. Bio Protoc. 3 (63), 374. 10.21769/bioprotoc.374 PMC4943750 27430005

[B28] Le Ouay B. Stellacci F. (2015). Antibacterial Activity of Silver Nanoparticles: A Surface Science Insight. Nano Today 10 (3), 339–354. 10.1016/j.nantod.2015.04.002

[B29] Liang L. Cui M. Zhang M. Zheng P. Deng Z. Gao S. (2015). Nanoparticles' Interference in the Evaluation of *In Vitro* Toxicity of Silver Nanoparticles. RSC Adv. 5 (82), 67327–67334. 10.1039/c5ra05863e

[B30] Liao C. Li Y. Tjong S. C. (2019). Bactericidal and Cytotoxic Properties of Silver Nanoparticles. Int. J. Mol. Sci. 20 (2). 10.3390/ijms20020449 PMC6359645 30669621

[B31] Maciag T. HooverStemerman G. A. M. B. Stemerman M. B. Weinstein R. (1981). Serial Propagation of Human Endothelial Cells *In Vitro* . J. Cel Biol 91, 420–426. 10.1083/jcb.91.2.420 PMC2111986 7309790

[B32] Matuszak J. Baumgartner J. Zaloga J. Juenet M. da Silva A. E. Franke D. (2016). Nanoparticles for Intravascular Applications: Physicochemical Characterization and Cytotoxicity Testing. Nanomedicine (Lond) 11 (6), 597–616. 10.2217/nnm.15.216 27003004

[B33] Medina C. Santos-Martinez M. J. Radomski A. Corrigan O. I. Radomski M. W. (2007). Nanoparticles: Pharmacological and Toxicological Significance. Br. J. Pharmacol. 150 (5), 552–558. 10.1038/sj.bjp.0707130 17245366 PMC2189773

[B34] Medina-Leyte D. J. Domínguez-Pérez M. Mercado I. Villarreal-Molina M. T. Jacobo-Albavera L. (2020). Use of Human Umbilical Vein Endothelial Cells (HUVEC) as a Model to Study Cardiovascular Disease: A Review. Appl. Sci. 10 (January), 938. 10.3390/app10030938

[B35] Melchinger H. Jain K. Tyagi T. Hwa J. (2019). Role of Platelet Mitochondria: Life in a Nucleus-free Zone. Front. Cardiovasc. Med. 6 (October), 1–11. 10.3389/fcvm.2019.00153 31737646 PMC6828734

[B36] Mirzajani F. Ghassempour A. Aliahmadi A. Esmaeili M. A. (2011). Antibacterial Effect of Silver Nanoparticles on Staphylococcus Aureus. Res. Microbiol. 162 (5), 542–549. 10.1016/j.resmic.2011.04.009 21530652

[B37] Niska K. Knap N. Kędzia A. Jaskiewicz M. Kamysz W. Inkielewicz-Stepniak I. (2016). Capping Agent-dependent Toxicity and Antimicrobial Activity of Silver Nanoparticles: An *In Vitro* Study. Concerns about Potential Application in Dental Practice. Int. J. Med. Sci. 13 (10), 772–782. 10.7150/ijms.16011 27766027 PMC5069413

[B38] Prakash Jai. Kaith B. S. Sun Shuhui. Bellucci Stefano. Swart Hendrik. C. (2019). Microbial Nanobionics. Nanotechnology Life Sci. 1. 10.1007/978-3-030-16534-5

[B39] Pritchett J. C. Naesens L. Montoya J. (2014). “Treating HHV-6 Infections,” in Uman Herpesviruses HHV-6A, HHV-6B, and HHV-7. Third Edition (Amsterdam, Netherlands: Elsevier), 311–331. 10.1016/B978-0-444-62703-2.00019-7 H

[B40] Qing Y. Cheng L. Li R. Liu G. Zhang Y. Tang X. (2018). Potential Antibacterial Mechanism of Silver Nanoparticles and the Optimization of Orthopedic Implants by Advanced Modification Technologies. Int. J. Nanomedicine 13, 3311–3327. 10.2147/IJN.S165125 29892194 PMC5993028

[B41] Radomski M. Moncada S. (1983). An Improved Method for Washing of Human Platelets with Prostacyclin. Thromb. Res. 30 (4), 383–389. 10.1016/0049-3848(83)90230-X 6351341

[B42] Rani K. Usha. (2017). Nanomedicine History of Nanomedicine. JNPE 3 (2January 2017), 37–40.

[B43] Raval N. Maheshwari R. Kalyane D. Youngren-Ortiz S. R. Chougule M. B. Tekade R. K. (2019). Importance of Physicochemical Characterization of Nanoparticles in Pharmaceutical Product Development. Basic Fundamentals of Drug Delivery. (Amsterdam, Netherlands: Elsevier), 369–400. 10.1016/B978-0-12-817909-3.00010-8

[B44] Redza-Dutordoir M. Averill-Bates D. A. (2016). Activation of Apoptosis Signalling Pathways by Reactive Oxygen Species. Biochim. Biophys. Acta (Bba) - Mol. Cel Res. 1863 (12), 2977–2992. 10.1016/j.bbamcr.2016.09.012 27646922

[B45] Ruiz-Giardin J. M. Ochoa Chamorro I. Velázquez Ríos L. Jaqueti Aroca J. García Arata M. I. SanMartín López J. V. (2019). Blood Stream Infections Associated with Central and Peripheral Venous Catheters. BMC Infect. Dis. 19 (1), 1–9. 10.1186/s12879-019-4505-2 31615450 PMC6794764

[B46] Salehi B. Berkay Yılmaz Y. Antika G. Boyunegmez Tumer T. Fawzi Mahomoodally M. Lobine D. (2019). Insights on the Use of α-Lipoic Acid for Therapeutic Purposes. Biomolecules 9 (8), 1–25. 10.3390/biom9080356 PMC6723188 31405030

[B47] Shah H. Bosch W. Thompson K. M. Hellinger W. C. (2013). Intravascular Catheter-Related Bloodstream Infection. Neurohospitalist 3 (3), 144–151. 10.1177/1941874413476043 24167648 PMC3805442

[B48] Shurbaji S. G Anlar G. A Hussein E. Elzatahry A. C Yalcin H. (2020). Effect of Flow-Induced Shear Stress in Nanomaterial Uptake by Cells: Focus on Targeted Anti-cancer Therapy. Cancers (Basel) 12 (7), 1–16. 10.3390/cancers12071916 PMC7409087 32708521

[B49] Tait S. W. Green D. R. (2013). Mitochondrial Regulation of Cell Death. Cold Spring Harb Perspect. Biol. 5, a008706. 10.1101/cshperspect.a008706 24003207 PMC3753705

[B50] Turcu I. Zarafu I. Popa M. Chifiriuc M. C. Bleotu C. Culita D. (2017). Lipoic Acid Gold Nanoparticles Functionalized with Organic Compounds as Bioactive Materials. Nanomaterials (Basel) 7 (2), 43. 10.3390/nano7020043 PMC5333028 28336877

[B51] Uchendu C. Ambali S. F. Ayo J. O. Esievo K. A. N. Umosen A. J. (2014). Erythrocyte Osmotic Fragility and Lipid Peroxidation Following Chronic Co-exposure of Rats to Chlorpyrifos and Deltamethrin, and the Beneficial Effect of Alpha-Lipoic Acid. Toxicol. Rep. 1, 373–378. 10.1016/j.toxrep.2014.07.002 28962253 PMC5598365

[B52] Verhoeven Arthur. J. Verhaar Robin. Gouwerok Eric. G. W. De Korte Dirk. (2005). The Mitochondrial Membrane Potential in Human Platelets: A Sensitive Parameter for Platelet. Qual. Arthur” 45 (January), 82–89. 10.1111/j.1537-2995.2005.04023.x 15647022

[B53] Veronesi G. Deniaud A. Gallon T. Jouneau P. H. Villanova J. Delangle P. (2016). Visualization, Quantification and Coordination of Ag+ Ions Released from Silver Nanoparticles in Hepatocytes. Nanoscale 8 (38), 17012–17021. 10.1039/c6nr04381j 27722394

[B54] Wadhwa R. Aggarwal T. Thapliyal N. Kumar A. Priya, Yadav P. (2019). Red Blood Cells as an Efficient *In Vitro* Model for Evaluating the Efficacy of Metallic Nanoparticles. 3 Biotech. 9 (7), 1–15. 10.1007/s13205-019-1807-4 PMC6588668 31245243

[B55] Zhang S. Liang X. Gadd G. M. Zhao Q. (2021). A Sol-Gel Based Silver Nanoparticle/polytetrafluorethylene (AgNP/PTFE) Coating with Enhanced Antibacterial and Anti-corrosive Properties. Appl. Surf. Sci. 535, 147675. 10.1016/j.apsusc.2020.147675

[B56] Zielinska E. Tukaj C. Radomski M. W. Inkielewicz-Stepniak I. (2016). Molecular Mechanism of Silver Nanoparticles-Induced Human Osteoblast Cell Death: Protective Effect of Inducible Nitric Oxide Synthase Inhibitor. PLoS ONE 11 (10), e0164137–25. 10.1371/journal.pone.0164137 27716791 PMC5055295

[B57] Zielinska E. Zauszkiewicz-Pawlak A. Wojcik M. Inkielewicz-Stepniak I. (2018). Silver Nanoparticles of Different Sizes Induce a Mixed Type of Programmed Cell Death in Human Pancreatic Ductal Adenocarcinoma. Oncotarget 9 (4), 4675–4697. 10.18632/oncotarget.22563 29435134 PMC5797005

